# Sonic Hedgehog Improves Redifferentiation of Dedifferentiated Chondrocytes for Articular Cartilage Repair

**DOI:** 10.1371/journal.pone.0088550

**Published:** 2014-02-12

**Authors:** Lin Lin, Qi Shen, Tao Xue, Xiaoning Duan, Xin Fu, Changlong Yu

**Affiliations:** 1 Institute of Sports Medicine, Peking University Third Hospital, Beijing, People's Republic of China; 2 Institute of Urology, Peking University First Hospital, Beijing, People's Republic of China; Indiana University School of Medicine, United States of America

## Abstract

Sonic hedgehog (Shh) is involved in the induction of early cartilaginous differentiation of mesenchymal cells in the limb. We investigated whether Shh could promote redifferentiation of dedifferentiated chondrocytes and have a favorable effect on the regeneration of cartilage. Articular chondrocytes of rats were separated and cultured. The redifferentiation of dedifferentiated chondrocytes transfected with Shh was evaluated using monolayer and pellet culture system. The signaling molecules (Ptc 1, Gli 1 and Sox9) of the hedgehog pathway were investigated. A rat model of articular cartilage defect was used to evaluate cartilage repair after transplantation with dedifferentiated chondrocytes. After Shh gene transfer, the hedgehog pathway was upregulated in dedifferentiated chondrocytes. Real time-PCR and western blot analysis verified the stronger expression of Ptc1, Gli1 and Sox9 in Shh transfected cells. Shh upregulates the Shh signaling pathway and multiple cytokines (bone morphogenetic protein 2 and insulin-like growth factor 1) in dedifferentiated chondrocytes. After transplantation in the joint, histologic analysis of the regenerative tissues revealed that significantly better cartilage repair in rats transplanted with Shh transfected cells. These data suggest that Shh could induce redifferentiation of dedifferentiated chondrocytes through up-regulating Shh signaling pathway, and have considerable therapeutic potential in cartilage repair.

## Introduction

The chondrocytes are highly specialized and responsible for the production and maintenance of the integrity of the cartilage extracellular matrix. It is well known that chondrocytes rapidly dedifferentiate during proliferation in monolayer culture [1]. Dedifferentiated chondrocytes are able to redifferentiate toward the chondrogenic lineage and re-express the chondrocytes phenotype with suitable stimuli [2]–[4]. The biosynthetic profiles of dedifferentiated chondrocytes resemble some of the phenotype displayed by osteoarthritic chondrocytes, and the matrix they produce is similar to that synthesized by chondroprogenitor cells [5]–[7]. Thus far, the mechanisms responsible for the regulation of these different chondrocyte-specific phenotypes are still poorly understood.

Chondrocytes modulate their pattern of gene expression in response to various growth factors. Currently, a new family of signaling proteins, the hedgehog (hh) family, is under investigation. It has been shown that the hedgehog signaling pathway is essential for the development of diverse tissues during embryogenesis [8]. In vertebrates, it is involved in the control of left-right asymmetry, organogenesis, spermatogenesis, and chondrogenesis. It is thought that hh signaling from prehypertrophic chondrocytes controls chondrocyte maturation [9], [10]. In normal cartilage development in vivo, sonic hedgehog protein (Shh) plays a critical role in the induction of early chondrogenic differentiation. Shh is expressed in the embryo by the floor plate and the notochord as well as in the limb bud and is thought to exert its control over skeletal patterning by inducing expression of downstream mediators, including BMPs and fibroblast growth factors (FGFs) [11]–[13]. Shh has a positive effect on matrix formation, as shown by Kellner et al. They found that chondrocytes produced up to 2.7 fold more extracellular matrix components than control constructs when grown in the presence of hedgehog proteins in a three dimensional scaffold [14]. Hedgehog proteins have the ability to promote differentiation of chondrogenic precursor cells in an ectopic cartilage formation model and their action in this process can be influenced and modified by synergistic or antagonist cofactors [16]. It remains unknown whether Shh can promote redifferentiation of dedifferentiated chondrocytes. What will happen after transplantation of chondrocytes expressing Shh in a load-bearing joint needs further evaluate.

The aim of this study is to investigate the effect of Shh on the redifferentiation of dedifferentiated chondrocytes and the regeneration of cartilage. Furthermore, we attempt to outline the molecular process of redifferentiation by over-expressing Shh.

## Materials and Methods

### Rat chondrocytes culture

Articular chondrocytes were isolated from femoral condyles of Lewis rats (male, 8w). Chondrocytes were isolated by digestion with 0.2% type II collagenase and resuspended in Dulbecco's modified Eagle's medium/F12 (DMEM/F12; Gibco, Laboratories, Grand Island, NY, USA) with 100 units/ml penicillin and 100 units/ml streptomycin. Chondrocytes were plated in tissue culture flasks at 10^4^ cells/cm^2^ and cultured in DMEM/F12 supplemented with 10% heat-inactivated fetal bovine serum (FBS; Gibco, USA) at 37°C in a humidified atmosphere of 5% carbon dioxide and 95% air. After approximately 10 days, when cells were subconfluent, primary cells (P0) were detached by treatment with 0.25% trypsin/1 mM EDTA, and plated at 5×10^3^ cells/cm^2^. Chondrocytes were induced to redifferentiate after fifth passages (P5) of expansion as described below.

### Transfection of dedifferentiated chondrocytes and monolayer cell culture

In vitro studies were based on optimized transfection of rat articular chondrocytes using the nonliposomal lipid formulation FuGENE 6 (Roche, Mannheim, Germany) essentially as previously described [17]. Chondrocytes were transfected with endotoxin-free expression plasmid vectors pcDNA3.1 carrying a human Shh cDNA (gift of Tabin Clifford) or enhanced green fluorescence protein (EGFP) gene (gift of Chunyan Zhou). Brie fly, plasmid DNA was complexed with FuGENE 6 in Opti-MEM and transferred to subcon fluent chondrocyte monolayers. Cells were treated with 4 U/ml bovine testicular hyaluronidase 12 h before and during transfection. The transduction efficiency by Fugene 6 was evaluated under fluorescence microscope. The transduction efficiency was 32.9±6.5%.

Fifth-passage chondrocytes were plated at a density of 1×10^4^ on culture slides. Twenty-four hours later cells were transfected with Shh and cultured in differentiation medium (DMEM supplemented with dexamethasone, sodium pyruvate, ascorbate-2-phosphate, proline, insulin, transferrin, l-glutamine, and 1% penicillin/streptomycin).

### Pellet cell culture and histological analysis

Cell pellets were made with fifth-passage chondrocytes transduced with pcDNA3.1-Shh or pcDNA3.1-GFP, as previously described [6]. The cells were trypsinized and 2×10^5^ cells in 0.5 ml of differentiation medium mentioned above were centrifuged at 500×g in 15-ml polypropylene conical tubes. The pellets were incubated at 37°C in 5% carbon dioxide, and the medium was changed every 3 days.

Pellets were harvested after 2 weeks, fixed overnight in 10% buffered formalin, dehydrated, and embedded in paraffin. After sectioning, the 5-ìm-thick pellet sections were deparaffinized and stained with toluidine blue. The expression of type II collagen in the pellets was analyzed by immunohistological staining. Sections were dewaxed in xylene, hydrated through graded alcohols. Endogenous peroxidase activity was blocked with 0.3% hydrogen peroxide in PBS. After blocking with goat serum (1∶100), sections were incubated with antibody against rat type II collagen (ab79013, Abcam Limited, Cambridge, UK) with dilution 1∶100 for 1 hr at 37°C. Control sections were incubated in PBS without the primary antibody. After washing three times, secondary anti-mouse IgG was added and incubated for 1 hr at 37°C. The colorization developed in DAB solution and counterstained by hematoxylin.

### Transplantation to articular cartilage defects in vivo

All animal experimental protocols were approved by the Animal Care and Use Committee of Peking University and conformed to National Institutes of Health Guidelines. Twenty-eight Lewis rats (12-week-old, body weights of 180–240 mg) were used in this study. In two groups of animals, the defects were created in both knee joints. In group I (8 animals), the defects were created in both knees in each animal and no cells were transplanted. 4 animals were killed after 4 weeks and 4 animals were killed after 8 weeks. In group II (18 animals), the right knee was transplanted with Shh transfected cells, and the left knee was transplanted with non transfected cells. Two rats were sacrificed 2 weeks after chondrocytes transplantation to evaluate the expression of Shh. The other animals were killed at 4 weeks or 8 weeks post-operation (8 animals at each time point).

The animal model of cartilage defect was performed as previously described by Gelse et al [16]. In brief, after anesthesia, the knee joint was exposed by a medial parapatellar incision and the patella was displaced laterally. Six lesions in the patellar groove of the femur were created by transverse movement of 27-gauge needle clamped between 2 metal spacers limiting the length of the tip to 0.3 mm. The exposed cartilage surface was treated for 20 minutes with PBS containing 0.1% hyaluronidase (Roche) and then washed thoroughly with PBS and completely dried using sterile cotton.

Chondrocytes cultured in monolayer were transplanted 48 h after transfection. 5×10^5^ cells in 6–8 µl medium containing serum in each defect of each group were loaded on top of the defects. The cells were left in the defect for 15 minutes to allow the cells to permeate the wound. And then 10 µl fibrin glue was added to cover the area of defects, and left for 5 minutes to adhere and to form a gel before suturing. Mediums without cells were injected as control. The joint capsule and the skin were closed with absorbable 5–0 sutures. After surgery, the rats were allowed to move freely within their cages.

### Histological evaluation of repaired cartilage

Rats were killed 4 or 8 weeks after injecting the cells. After sacrifice, the femoral condyles were fixed in formalin, and then decalcified and embedded in paraffin. Coronal sections (6 µm thick) were obtained from the defects and were stained with hematoxylin-eosin (HE), toluidine blue for microscopic examination of the repaired tissue. Sections from each animal were randomized to eliminate bias, and then examined and scored independently by three independent researchers. Samples were graded according to a histological scale which is described previously [4]. The expression of type II collagen in the regenerated tissues was analyzed by immunohistological staining. Four samples were stained with immunohistological staining to assess Shh expression. Staining for Shh was performed using the similar protocol described for the type II collagen mentioned above. The dilution of the primary antibody Shh (specific for human, ab73958, Abcam Limited, Cambridge, UK) was 1∶50.

### Statistical analysis

All data are expressed as the mean ± SD (standard deviation). The differences in the mean values between the different groups were compared with the nonparametric Mann – Whitney U-test using SPSS (v12.0) statistical software (SPSS Inc., Chicago, IL). P-Values less than 0.05 were considered significant.

## Results

### Monolayer culture multiplication of chondrocytes leads to dedifferentiation

The typical round chondrocytes morphologically transformed into flattened fibroblast-like cells after serially passage. The expression of type II collagen, aggrecan was significantly decreased and was almost not found in P5, while type I collagen expression was significantly increased as confirmed by real time RT-PCR and immunohistological staining (data not shown). The results were consistent with our previous study [4]. So the chondrocytes of P5 was chosen to do the subsequent experiments.

### Redifferentiation of dedifferentiated chondrocytes stimulated by Shh in pellet culture

**Figure 1:**
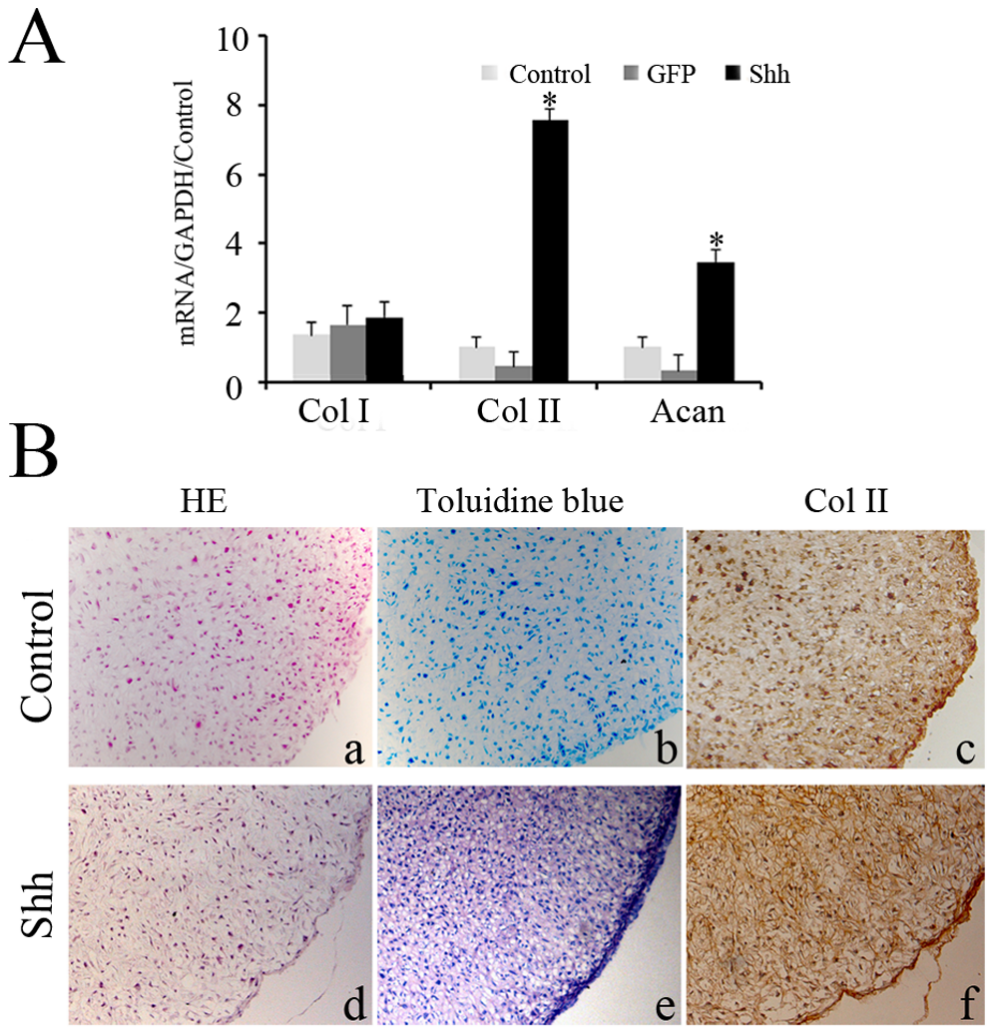
Redifferentiation of dedifferentiated chondrocytes stimulated by Shh in monolayer and pellet culture. RT-PCR revealed that the expression of type II collagen and aggrecan in Shh transfected cells after 4 days in monolayer culture in differentiation medium (A). The matrix of Shh transfected chondrocytes was toluidine blue positive after 14 days in the pellet cultured in differentiation medium, and chondrocytes-like cells were found in the pellet. Type II collagen expression was proved by immunological staining (B). Original magnification 10×. *p<0.05

Real time RT-PCR and western blot analysis clearly demonstrated the expression of Shh in Shh transfected cells at 48 h but not in GFP transfected and untransfected cells (data not shown). When cultured in differentiation medium, the expression of type II collagen and aggrecan was upregulated in shh transfected cells proved by real time RT-PCR, while the expression of type II collagen in P5 chondrocytes transduction with GFP and the control cells was very little (Figure 1A). P5 chondrocytes transfection with GFP failed to form compact pellets and toluidine blue staining was very weak. However, P5 chondrocytes transfection with Shh formed very compact pellets that contained chondrocytes-like cells surrounded by toluidine blue-positive matrix, and type II collagen expression was confirmed by immunological staining (Figure 1B).

### Shh upregulates the Shh signaling pathway and multiple cytokines in dedifferentiated chondrocytes

**Figure 2:**
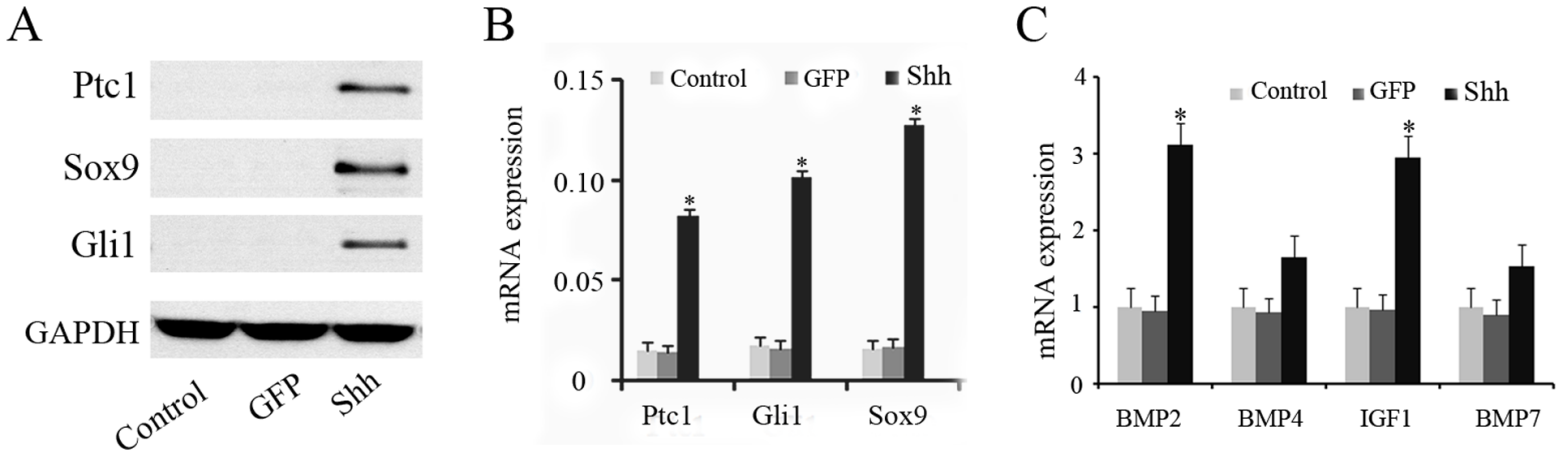
Shh upregulates the Shh signaling pathway and multiple cytokines in Shh transfected chondrocytes. To explore potential mechanisms responsible for the redifferentiation effect of Shh, we evaluated the Shh signaling pathway. Real time RT-PCR and western blot analysis verified the stronger expression of Ptc1, Gli1 and Sox9 in Shh transfected cells after 48 hours (A, B). The mRNA expression of BMP2 and IGF1 were upregulated by Shh after 48 hours (C). *p<0.05.

To explore potential mechanisms responsible for the redifferentiation effect of Shh, we evaluated the Shh signaling pathway. Real time RT-PCR and western blot analysis verified the stronger expression of Ptc1, Gli1 and Sox9 in Shh transfected cells after 48 hours (Figure 2A, B). Furthermore, we investigated the mRNA expression of candidate genes involved in the redifferentiation of chondrocytes. Two of the investigated genes (BMP2 and IGF1) were upregulated by Shh (Figure 2C).

### Shh expression analysis in vivo

**Figure 3:**
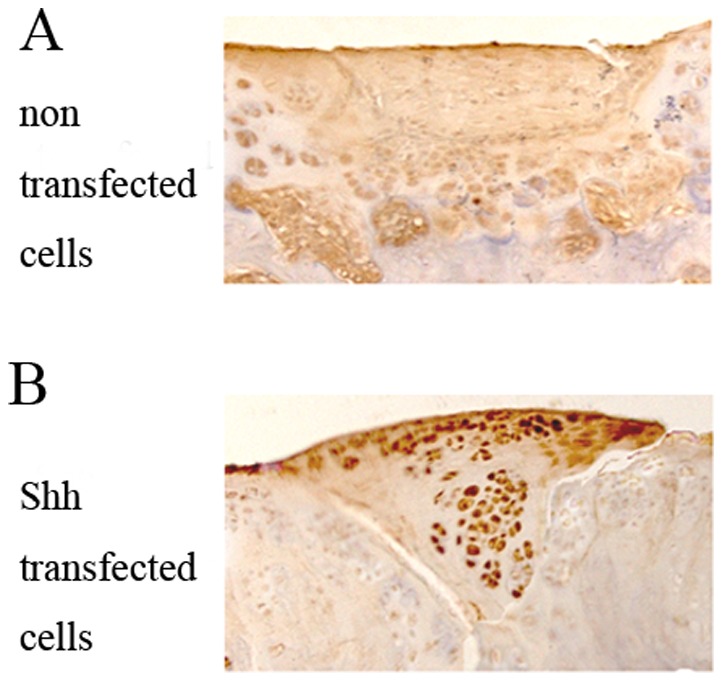
Shh expression in vivo. In non-transfected group, Shh expression was not detected (A). In the defects in Shh group, the repair tissues contained Shh protein proved by immunohistochemical staining 2 weeks after transplantation (B). In the negative control without primary antibody, Shh expression was not detected (data not shown). Original magnification 20×.

In the defects in implanted with Shh-transfected cells group, the repair tissues contained Shh protein proved by immunohistochemical staining after transplantation (Figure 3). In non-transfected group, Shh expression was not detected. In the negative control without primary antibody, Shh expression was not detected (data not shown).

### Improvement in repair of cartilage defects by Shh in rat model

The animals were sacrificed at 4 or 8 weeks after the transplantation, and all knee joints were histologically analyzed. Toluidine blue staining and HE staining allowed for morphologic assessment of the cartilage. Parallel sections were additionally analyzed by immunohistochemistry for type II collagen. In all samples, the underlying bone stained negative for type II collagen and served as an internal control. Type II collagen was detected in intact hyaline articular cartilage and in calcified cartilage. The cartilage regeneration in the treated defects at 4 and 8 weeks was evaluated for filling of the defect, integration of the repair tissue into adjacent preexisting tissue and proteoglycan staining of the repair tissue.

**Figure 4:**
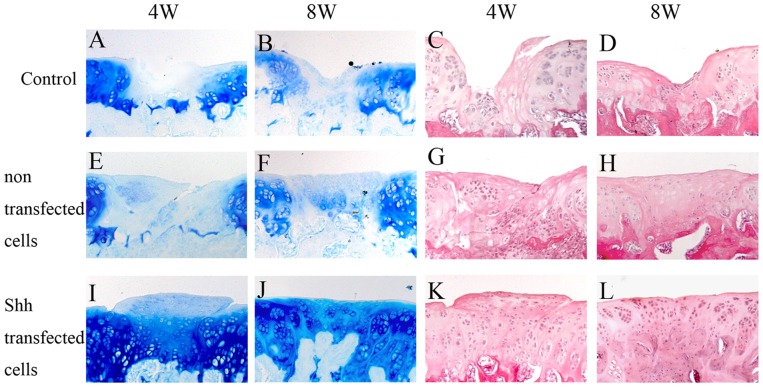
Histologic evaluation of the regeneration of cartilage 4 weeks and 8 weeks after transplantation. Representative results of histologic evaluation of the regenerative tissues stained with toluidine blue: control group (A, B), non-transfected group (E, F) and Shh group (I, J); hematoxylin-eosin: control group (C, D), non-transfected group (G, H) and Shh group (K, L). Original magnification 20×. 4w: 4weeks, 8w: 8weeks.

**Figure 5:**
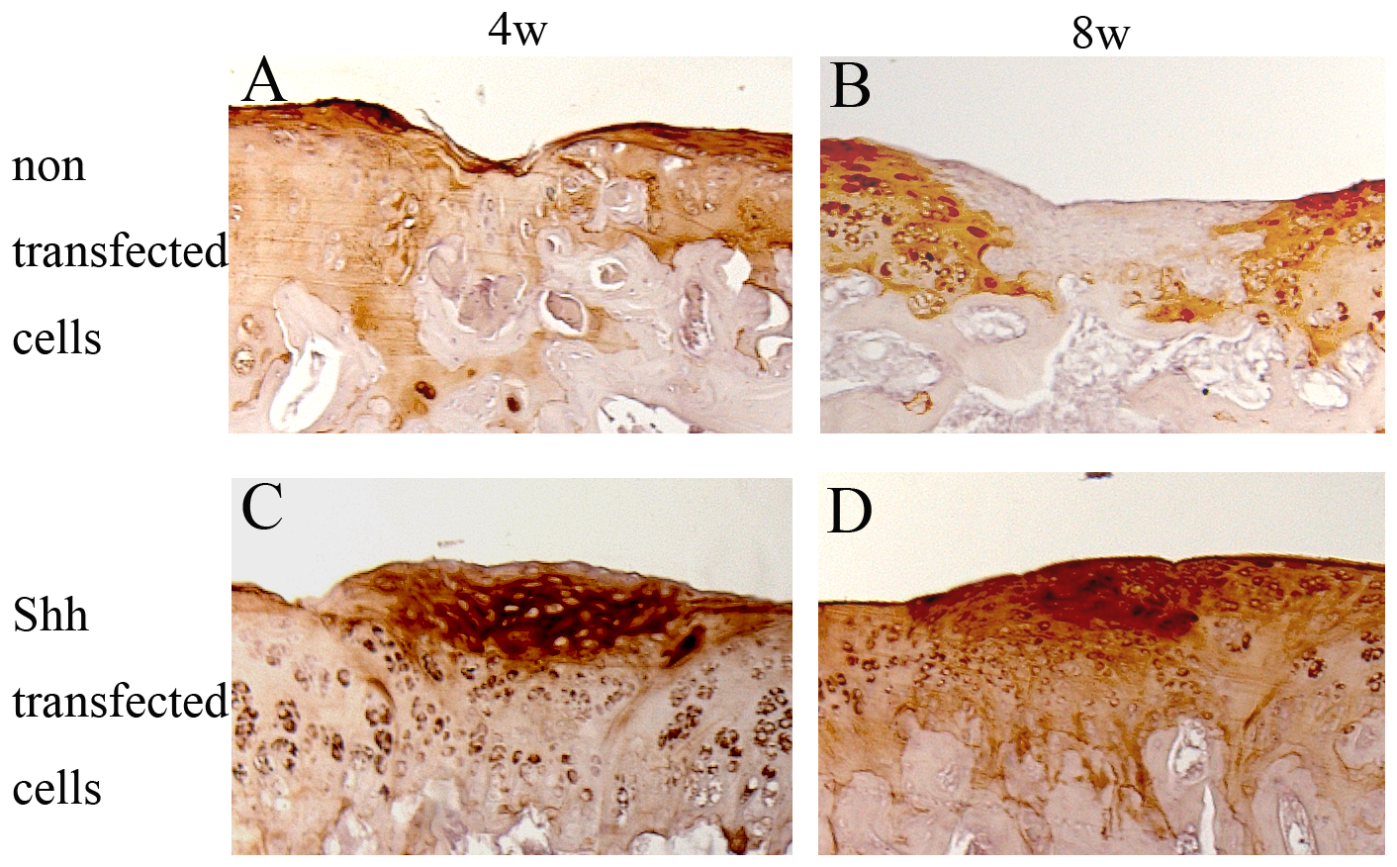
Representative results of immunohistological staining for type II collagen of repaired tissues after transplantations in treatment group. Immunohistological staining of regenerative tissues 4 weeks after transplantation of non-transfected chontrocytes (A), and Shh transduced chondrocytes (C). Regenerative tissues 8 weeks after transplantation with immunohistological staining: non-transfected chondrocytes (B), and Shh transfected chondrocytes (D). Original magnification 20×. 4w: 4weeks, 8w: 8weeks.

At 4 and 8 weeks, the defects in the articular cartilage did not heal spontaneously. Untreated fissures were found either empty or contained cells and matrix debris (Figure 4A, B, C, D). The bordering wall of the defects showed a decrease in toluidine blue staining, indicating a loss of proteoglycans in this region (Figure 4A, B). Joint regions, e.g., femoral condyles and tibia plateau, which had not been manipulated mechanically showed an intact articular cartilage. Transplantation of non-transfected cells also resulted in only insufficient repair. The repair tissue consisted of fibrous mesenchyme with fading toluidine blue staining indicated that anabolic stimulation occured. Also the repair tissue appeared not well integrated with the surrounding articular cartilage at 4 and 8 weeks (Figure 4E, F, G. H). The cells failed to fill up the lesions and produce a cartilage-like matrix that was negative for type II collagen with a fibroblast-like phenotype (Figure 5A, B).

At 4 and 8 weeks in the shh group, round chondrocytes cells were present within the matrix which was stained strongly by toluidine blue. Although the cell arrangement differed from normal cartilage, the staining of toluidine blue indicated a proteoglycan-rich matrix (Figure 4I, J). The newly formed cartilage showed clearly positive type II collagen staining (Figure 5C, D). In the majority of the defects, the repair tissue was well integrated, leaving no clefts between repair tissue and preexisting cartilage on either side of the defect, and restored a continuous, albeit not always regular, cartilage surface (Figure 4K, L). Adjacent regions of preexisting cartilage did not show any loss of proteoglycans, which indicated a paracrine effect on neighboring chondrocytes.

Reparing of articular cartilage was improved histologically in vivo at 8 weeks compared with that at 4 weeks. The grading scores for control group, non-transfected group and Shh group are 2.9±0.6, 6.9±1.1 and 15.7±1.9 respectively 4 weeks after transplantation, and are 4.3±0.8, 8.5±1.7 and 21.1±2.1 respectively 8 weeks after transplantation.

## Discussion

The Hedgehog gene was identified in Drosophila as a crucial regulator of cell-fate determination during embryogenesis. There are three known mammalian Hedgehog family members: Sonic hedgehog (Shh), Desert hedgehog (Dhh) and Indian hedgehog (Ihh). Among them, Shh is the most widely expressed during development and is the best studied [18], [19] Hedgehog signaling occurs through the interaction of Hedgehog ligand with its receptor, Ptc1. Once Hedgehog binds its receptor, subsequent activation of Smoothened (Smo) initiates signaling events that lead to the regulation of transcriptional factors belonging to the Gli family, which modify the expression of downstream target genes [20], [21]. Sox9 is an important chondrocyte differentiation factor and is expressed in all cartilaginous tissues. Furthermore, Sox9 has been shown to bind to the promoters of the chondrocyte differentiation markers, collagen II and aggrecan, and activate their transcription. Cells lacking Sox9 are unable to enter the cartilage differentiation program [22]. Here we showed that the expression of Sox9 was upregulated by Shh in dedifferentiated chondrocytes, thus leading to the redifferentiation of chondrocytes.

Previous studies have shown that exogenously added Shh is sufficient to initiate the entire chondrogenic program in explanted chick presomitic mesoderm [13]. Also, they found that Shh treatment induced, after a lag period, endogenous BMP expression that completed the chondrogenic differentiation process. The chondrogenic effect of hedgehog proteins on MSCs seems to be mediated on one hand by their direct induction of Sox9. On the other hand, the hedgehog mediator Gli binds to BMP promoters [23], [24]. The findings of Zeng et al. suggested that Shh and BMP signals worked in sequence, and that Sox9 subsequently initiated the chondrocyte differentiation program in a variety of cellular environments [22]. Similar to MSCs, dedifferentiated chondrocytes appear to be multipotent mesenchymal cells and can enter myogenic, osteogenic and adipogenic differentiation throughout passaging in vitro [25]. Our results seem to indicate a similar role for Shh in dedifferentiated chondrocytes.

Recently, studies of Koelling et al. show that repair tissue from human articular cartilage during the late stages of osteoarthritis harbors a unique progenitor cell population, termed chondrogenic progenitor cells (CPCs) [26], [27]. These cells exhibit stem cell characteristics such as clonogenicity, multipotency, and migratory activity. The isolated CPCs, which exhibit a high chondrogenic potential, were shown to populate diseased tissue ex vivo. The redifferentiation observed may be explained by the reprogramming of differentiated chondrocytes, and/or by the existence of a separate undifferentiated, multipotent cell population within articular cartilage. Thus far, it is not clear whether the redifferentiation phenomena are due to phenotypic plasticity of articular chondrocytes or the existence of progenitor cells within adult articular cartilage.

We transplanted the cells into the defects of cartilage to evaluate the effect of Shh on the redifferentiation of the dedifferentiated chondrocytes in the complex biological and physical environment of load-bearing joint. After transplantation of dedifferentiated chondrocytes, chondrocytes in the regenerative tissues displayed Shh-positive with immunohistological staining, and proteoglycan-rich matrix was found around the chondrocytes proved by toluidine blue stain. These findings indicate that the transplanted cells recovered chondrocytes-like phenotype and participated in the repair of articular cartilage.

Hedgehog signaling is activated in osteoarthritis, and higher levels of hedgehog signaling in articular chondrocytes cause a more severe osteoarthritis phenotype [28]. The investigators utilized transgenic mice, a model in which the activation of hedgehog signaling or the doses of hedgehog proteins were very high. The action of hedgehog proteins on normal articular chondrocytes is unclear. Kellner et al. found that Shh has a positive effect on matrix formation of chondrocytes [14]. Iwakura et al. found that Shh and Ihh signaling has a stimulatory effect on superficial zone protein accumulation in surface zone cartilage and isolated articular chondrocytes [29]. In this study, we found that the expression of Ptc1and Gli1 in Shh transfected cells. Subsequent endogenous BMP expression is obligate for maintaining Sox9 expression and complete chondrogenic redifferentiation.

Warzecha et al. found that Shh protein could promote proliferation and chondrogenic differentiation of bone marrow derived mesenchymal stem cells in vitro [30]. Growth factors such as BMP-2 and IGF-1 have been shown to promote the chondrogenic differentiation of mesenchymal stem cells, the synthesis and deposition of extracellular matrix components by chondrocytes as well as cell proliferation [16]. In cell culture, both factors suppress chondrocyte dedifferentiation and stabilize the chondrocyte phenotype [16], [31]. In this study, we found that Shh stimulates the expression of BMP-2 and IGF-1during the redifferentiation of dedifferentiated chondrocytes. IGF-1 has been shown to protect a variety of cells against apoptosis. We observed that Shh upregulates the mRNA expression of IGF1 in dedifferentiated chondrocytes. These data suggested that Shh may exert both direct and indirect effects to promote cartilage repair.

In summary, our data indicate that Shh could induce redifferentiation of dedifferentiated chondrocytes by up-regulating hedge signaling, and have considerable therapeutic potential in cartilage repair.

## Supporting Information

Table S1 (DOC)

Method S1 (DOC)
