# Supplementary material for: Sonic Hedgehog Improves Redifferentiation of Dedifferentiated Chondrocytes for Articular Cartilage Repair
Source: PLoS One. 2014 Feb 12;9(2):e88550. doi: 10.1371/journal.pone.0088550 (PMC3922882; doi:10.1371/journal.pone.0088550)
Supplement: Table S1 — (DOC) [file pone.0088550.s001.doc]

**Table S1. Specific primers used for real-time RT-PCR**

| Gene | Primer Sequence | Ta* (℃) |
| --- | --- | --- |
| Shh | 5'-AGGGCTGGGACGAAGATGG-3'  5'-GCTTTCACCGAGCAGTGGATA-3' | 58 |
| aggrecan | 5'-AGTCTACCCAGCACCCTAC-3'  5'-TGTTTCTCCTGACCCTTCT-3' | 57 |
| Collagen II | 5'-ACCTGAAACTCTGCCACCC-3'  5'-CTTGCTCTTGCTGCTCCAC-3' | 56 |
| Collagen I | 5'-GGCAACCTCAAGAAGTCCC-3'  5'-GTGCAGCCATCCACAAGC-3' | 58 |
| Sox9 | 5'- AGCCCTGGTTTCGTTCT-3'  5'- CTGCTCGTCGGTCATCTT-3' | 55 |
| Ptc1 | 5′-ATGCTGGAGGAGAACAAGCAA-3′,  5′-CGAGGACCCCATCATCAGAT-3′ | 53 |
| Gli1 | 5′- GACCGGTTCTGTCATGTCG-3′  5′-ACCTGGTTCATCATCACTAACAC-3′ | 59 |
| GAPDH | 5'- GCTGGGGCTCACCTGAAGGG-3'  5'- GGATGACCTTGCCCACAGCC-3' | 55 |

*Ta: temperature of annealing
