# Supplementary material for: Sonic Hedgehog Improves Redifferentiation of Dedifferentiated Chondrocytes for Articular Cartilage Repair
Source: PLoS One. 2014 Feb 12;9(2):e88550. doi: 10.1371/journal.pone.0088550 (PMC3922882; doi:10.1371/journal.pone.0088550)
Supplement: Method S1 — (DOC) [file pone.0088550.s002.doc]

**Method S1**

**Quantitative real-time RT-PCR analysis.** Total RNA was harvested using TRIzol (Invitrogen) according to manufacturer’s instructions, and then quantified by ultraviolet spectroscopy at assigned time points post-induction. cDNA synthesis was performed using total RNA (1 μg) as a template by oligo(dT) priming using the Superscript First Strand Synthesis System for RT-PCR (Invitrogen). Real-time RT-PCR was performed with an optional continuous fluorescence detection system (MJ research, MA, USA). 1 μl of reverse transcribed product and 1×SYBR green (Molecular Probes, Eugene, USA) were included in 25 μl reaction mixture (10 mM Tris-HCL, pH 8.3, 50 mM KCL, 1.5 mM MgCl2, 200 μM of dNTP mix, 0.2 μM of each primer and 1 unit of Taq DNA polymerase). Oligonucleotide primers (table 1) were designed using Oligo 6 primer analysis software. Each cycle consisted of 1 min denaturation at 94 °C, 1 min annealing at 57 °C and 1 min extension at 72 °C. mRNA levels were normalized to GAPDH using the comparative cycle threshold (CT) method.

**Western blotting analysis**. The harvested medium was collected on the indicated days. Protein concentration was measured by BCA protein assay kit (Pierce Biotechnology, Inc., Rockford, IL, USA) using bovine serum albumin as the standard. Proteins were run on SDS-PAGE gels and electrotransferred to nitrocellulose membrane at 4°C for 2 h. The blots were probed with anti-Ptc1, Sox9 and Gli1 (Abcam Limited, Cambridge, UK) at 1:500 dilutions overnight at 4°C. The proteins were detected by chemiluminescence according to manufacture’s recommendations (ECL, Amersham Arlington Heights, IL, USA). GAPDH was used as an internal control.
